# The effect of particle shape on the deformation and stress reduction of a gravel soil due to wetting

**DOI:** 10.1038/s41598-021-95731-y

**Published:** 2021-08-16

**Authors:** Reza Mahinroosta, Vahid Oshtaghi

**Affiliations:** 1 School of Engineering, Faculty of Business, Justice and Behavioural Sciences, Charles Sturt University, Panorama Avenue, Bathurst, NSW 2795 Australia; 2 Institute for Land, Water and Society, Charles Sturt University, Bathurst, NSW Australia; 3 Civil Engineering Department, Zanjan University, Zanjan, Iran

**Keywords:** Civil engineering, Mechanical properties

## Abstract

This paper investigates the effect of particle shape on the stress reduction and collapse deformation of gravelly soil using a medium-scale direct shear test apparatus under different relative densities, normal stress, and shear stress levels. A new method based on the Micro-Deval test was introduced to produce sub-angular particles from angular particles. Therefore, two series of soil specimens were obtained with the same rock origin, particle size distribution, and relative density but different particle shapes. In addition to traditional direct shear tests on dry and wet specimens, a specific test procedure was applied to explore the stress reduction and collapse of soil specimens due to wetting. The results of the tests, including shear stress–shear displacement and vertical displacement-shear displacement, were compared. The results showed that the stress reduction and settlement due to wetting increased with vertical and shear stress levels in both types of particle shapes, with higher values in angular particle shapes. The particle breakage of the soil specimens was also studied quantitatively using the change in the particle size distribution before and after the test. It was shown that the wetting of the samples had more impact on the particle breakage in angular gravel than sub-angular gravel, which increased linearly with the normal stress.

## Introduction

Sudden deformation in granular material due to wetting is called collapse deformation. This usually occurs in recent soil deposits with a high void ratio^1^ and reservoir banks after impoundment^2^. In addition, soils in the foundation of structures or in the body of embankments may experience high volumetric deformation if they are poorly compacted. Laboratory tests and field instrumentation have shown the possibility of collapse behaviour in a variety of granular material from silt and sand to gravel and rockfill^3–7^. The collapse behaviour of coarse grain material in the upstream zone of embankment dams has a significant effect on the final deformation due to impoundment^8^. The deformation may result in internal erosion, an uncontrolled leak from an embankment dam or even a rupture of the dam due to a localised collapse^9^. Collapse settlement is also important in road construction^10^ and reclaimed lands on which buildings are to be constructed.

There are different reasons for the collapse deformation of granular material, such as a sudden reduction in the stress state due to the addition of water, easier sliding of the soil grains due to inter-particle strength reduction, particle breakage and change in the contact bonding mechanism^11^. In the case of particle breakage, different factors are responsible, such as the particle size, stress state, previous stress history, relative compaction, particle shapes, rock type and water itself^12,13^. In terms of the bonding mechanism, the water menisci play a significant role in the collapse of granular soils. Research showed that applying polar liquids different from water can reduce the collapse potential by 90%^14^. With a similar concept, pollutants in the water, e.g., from the leakage of sewage systems, also has a great impact on the surface collapse due to chemical attack to the bonds between the soil particles^15^.

Nobari and Duncan^16^ appear to have been the first researchers who studied the collapse phenomenon and stress reduction in granular materials subjected to wetting. They showed that the collapse deformation of the material was decreased by an increase in the relative density and moisture content. Alawaji^6^ showed that the collapse deformation of the soil increases with the compressibility of the material and the normal stress. Based on a discrete element modelling of a rockfill column, Silvani et al.^17^ concluded that due to the rearrangement of the soil particles during saturation, the soil matrix resulted in a significant reduction in the friction angle. Matric suction, a factor related to moisture content, is another principal factor affecting collapse deformation; the higher the matric suction, the higher the collapse potential for soil with specific density^7^. Shahriar et al.^18^ conducted a comprehensive literature review on the effect of water level on the settlements of the foundations on granular material. They found that the settlement of the foundations due to water level rise depended on the stress level, soil friction angle, and soil relative density, and was even more than twice the settlement of the foundation on dry granular material.

The shape of the particles is an important factor in the deformation characteristics of granular soils. Alonso^19^ showed that collapse settlements of embankment dams with alluvial gravels of rounded nature were very small, especially when well-graded materials were found in the alluvial deposits. The particle shape may depend on the origin of the soil mass and its weathering process. Soil particles located close to their origin are generally angular, while those carried by wind or water are usually rounded. Brink and van Rooy^20^ showed how a residual soil and transported soil with the same initial void ratio did not experience the same collapse settlement due to wetting. Shin and Santamarina^21^ conducted oedometer tests on several mixtures of round Ottawa sand and angular blasting sand. They showed that the oedometric compressibility of the soil mixture increased with the angularity of the soil. This means that the angularity of the soil increases the potential settlement of the soil mass subjected to a normal load. Similar results were shown by Cabalar et al.^22^ on four types of sands from Turkey and Cyprus; they found that material with higher roundness and sphericity in the particles showed lower compressibility.

The above review shows that minimal attention has been given to the effect of particle shape on the collapse deformation and stress reduction of gravel soils due to wetting. The prediction of the collapse settlement is of paramount importance in alluvial deposits or man-made geo-structures subjected to changes in groundwater conditions, such as embankment dams. In most of the previous studies on the effect of particle shape, different soils with different origins were investigated; however, in this study, a new method based on a standard laboratory technique is introduced to generate sub-rounded particles from angular particles so that particle shapes with the same mineral hardness are investigated.

This paper presents the outcomes of a series of 40 direct shear tests on the stress reduction and collapse deformation of gravel soil with angular and sub-angular particle shapes. While the effect of relative density is investigated in this study, the main focus is on particle shape, shear stress level and vertical stress on the collapse behaviour of the material. For all soil samples, index and physical properties were determined for the soil description and sample preparation. Soil specimens with sub-angular particles and angular particles of the exact origin were prepared with the same particle size distribution (PSD) and density. Then the medium-scale direct shear test was used to evaluate deformation and strength characteristics of the soil specimens in three states: dry, wet, and dry–wet.

### Soil material, sample preparation and method of testing

#### Soil material

**Figure 1:**
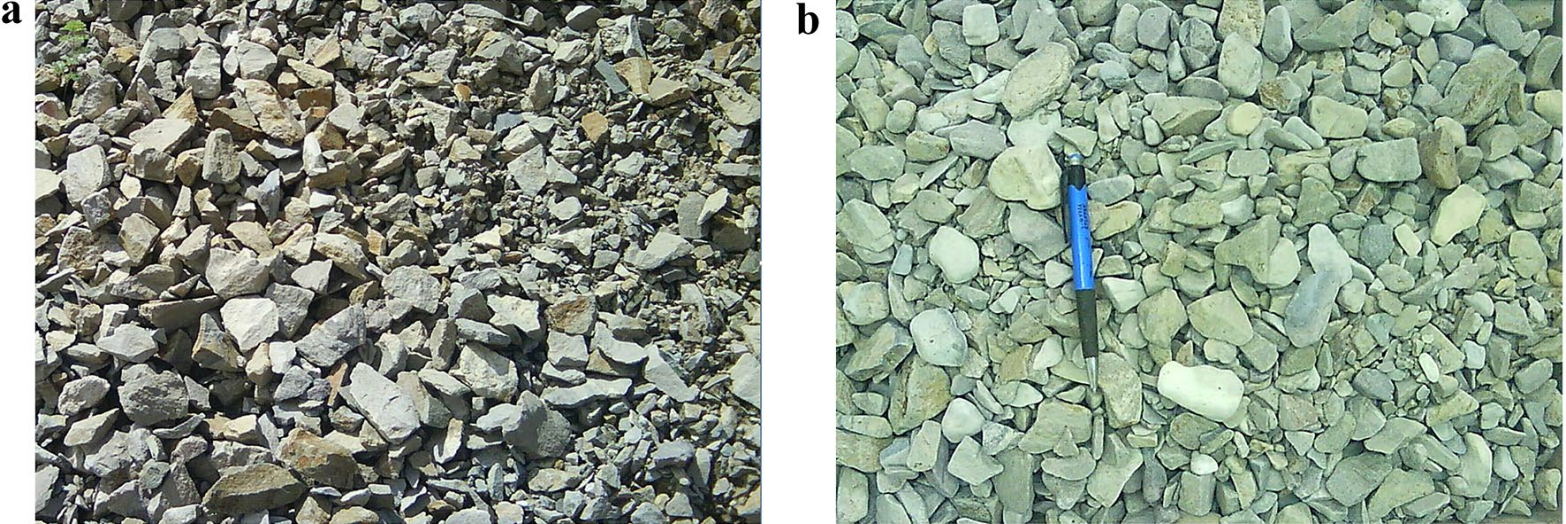
Soil particles. (**a**) Angular particle shapes. (**b**) Sub-angular particle shapes.

Soil samples in this study consisted of coarse aggregates obtained from the borrow resources of the ballast required for a railroad construction near Mianeh, a city in north west Iran. The rock type was Trachytic tuff with an average dry and saturated compressive strength of 117 and 51 MPa, respectively. The crushed particles (Fig. 1a) were the result of a controlled explosion of the quarry. The photos of the soil particles were used to quantify the particle shape based on the procedure recommended by Muszynski and Vitton^23^ to determine the angularity (or roundness) (A) and sphericity (S)^24^ of the particles. Angularity is the ratio of the average diameter of the corners and edges to the diameter of the maximum inscribed sphere in a particle. Sphericity is the ratio of the diameter of a minimum sphere circumscribing a gravel particle to the maximum inscribed sphere in that particle. The authors evaluated both A and S based on 100 individual particle images with different sizes from 5 to 25 mm. The quarter method of soil sampling was used to prepare 20 soil specimens from where the individual particles were selected. Based on the quantitative analysis of the data, the angularity of the particles was in the range of 0.12 and 0.23, with a median of 0.18. The sphericity of the particles varied from 0.36 to 0.74, with a median of 0.51. The average A and S in the material was 0.18(± 0.03) and 0.52(± 0.09), respectively. Therefore the soil particles were classified as “angular” based on the most widely used comparator^25,26^ in Table 1. The average angularity of the particles is towards the lower band of the angular particles (0.17–0.25).

**Table 1:** Classification of the soil particles based on their angularity^26^.

Angularity	Class limits	Geometric mid-point
Very angular	0.12–0.17	0.14
Angular	0.17–0.25	0.21
Sub-angular	0.25–0.35	0.30
Sub-rounded	0.35–0.49	0.41
Rounded	0.49–0.70	0.59
Well rounded	0.70–1.00	0.84

**Figure 2:**
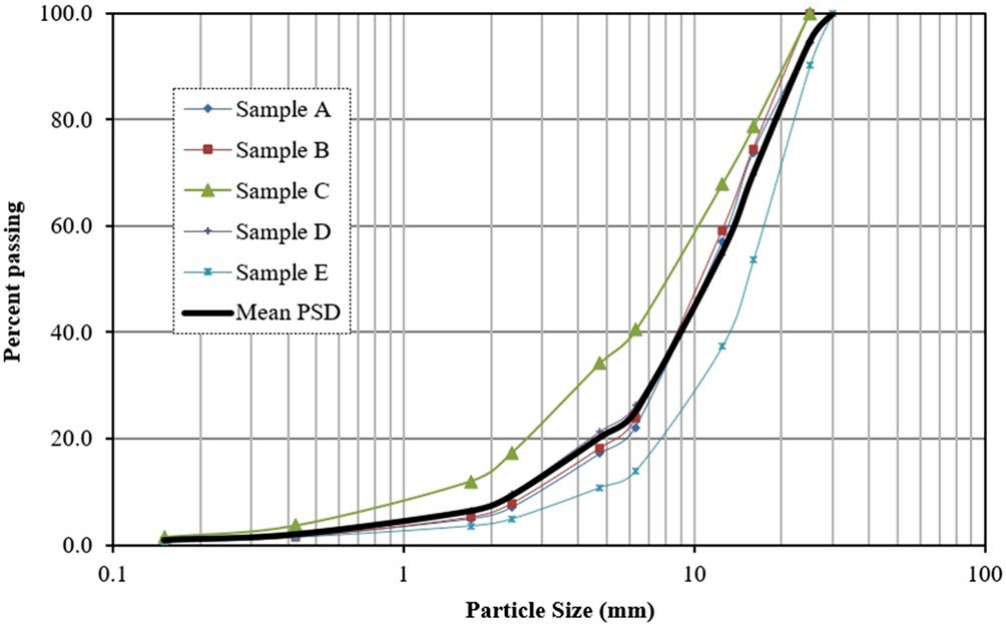
Particle size distribution of several samples and mean PSD used for preparing shear box specimens.

The particle sizes of five selected samples (sample A to sample E) are presented in Table 2. To conform to the requirements of the direct shear test suggested by ASTM D3080-11^27^ the maximum size of the particles was set to 30 mm (less than 1/10 of the shear box dimension). The fine contents of all samples are less than 1%. Sample A has the largest particles with more than 70% greater than 10 mm. Sample C has the smallest particles with around 40% greater than 10 mm. The PSD curves of five selected samples are shown in Fig. 2 compared to the mean PSD curve. In this study, the mean particle size distribution of all samples was used to prepare all the soil specimens for the direct shear tests. The particle sizes of the mean sample are also presented in Table 2. The mean sample has more than 55% greater than 10 mm. This mean sample is classified as well graded clean gravel (GW) based on the Unified Soil Classification System.

**Table 2:** Particle sizes and percent passing in each individual soil sample.

Particle size (mm)	Percent passing					Mean PSD
	Sample A	Sample B	Sample C	Sample D	Sample E	
30	100.0	100.0	100.0	100.0	100.0	100.0
25	94.5	100.0	100.0	94.6	90.2	94.9
16	73.8	74.5	78.6	69.7	53.7	70.1
12.5	57.0	59.1	67.9	54.6	37.4	55.2
6.3	22.2	23.8	40.5	26.2	14.0	25.3
4.75	17.2	18.3	34.2	21.3	10.8	20.4
2.36	7.1	7.9	17.4	9.6	4.9	9.4
1.7	4.9	5.3	12.0	6.4	3.6	6.4
0.425	1.7	1.5	3.7	2.0	1.5	2.1
0.15	0.9	0.8	1.5	0.9	0.7	1.0
0.075	0.5	0.4	1.0	0.6	0.5	0.6

#### Preparation of soils with sub-angular particles

**Figure 3:**
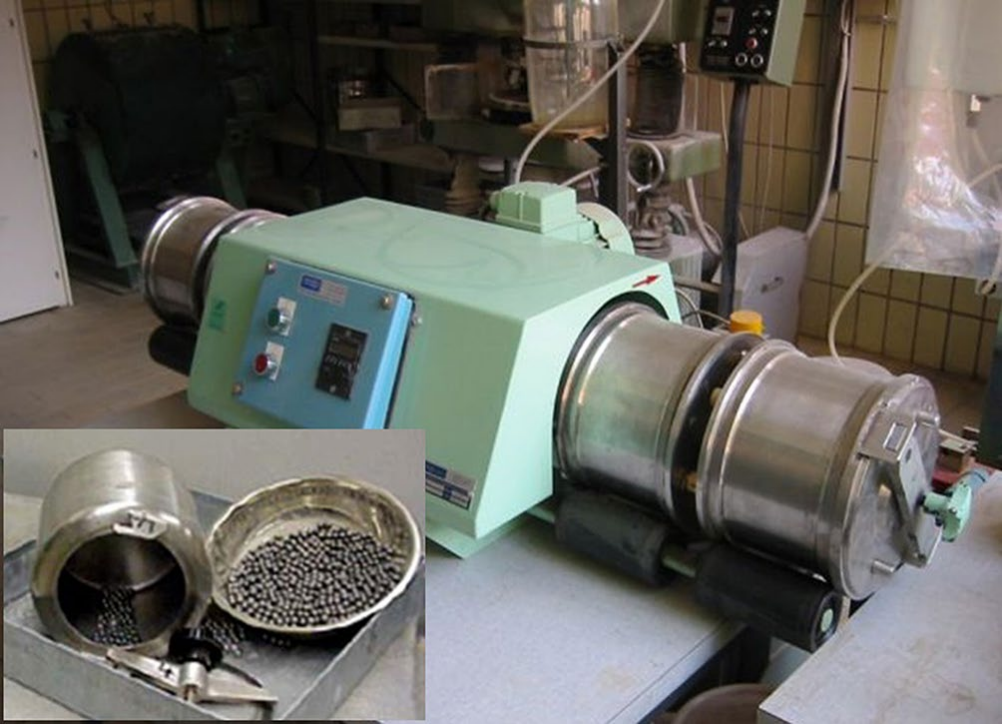
The Micro-Deval machine used in the study.

Because of the development process, weathering and the mechanism of soil deposits, it is not easy to find natural granular material with different particle shapes of the same origin. The Micro-Deval abrasion test^28^ was used (Fig. 3) for the preparation of the samples with sub-angular particle shapes. This test is usually used to determine the abrasion loss of fine grain aggregate and its durability in a smooth drum, including water and small stainless-steel balls. The equipment in this study was built by GENEQ (Geneq inc, Canada) with the jar speed of 100 rpm (± 5 rpm). The jar itself was a 5 L stainless steel container with locking cover.

In the standard method of conducting this test, 1500 g of coarse aggregate is used and immersed in tap water for a minimum of 1 h. Then, 5000 g of stainless-steel balls with a 9.5 mm diameter are added to the test sample by using a Micro-Deval machine to rotate the jar at 100 rpm for 2 h. The soil sample is washed over a specified sieve (1.18 mm in diameter for coarse aggregate), and the percentage of retained soil is determined by comparing the oven-dried mass of the retained sample to the original total weight of the sample. After conducting this test on several soil samples from chosen angular soil material, the authors noticed that the remaining particles were sub-angular. The impact of the small steel balls on the soil particles during the test smoothed the corners of the particles. Soil material after the abrasion test with Micro-Deval is shown in Fig. 1b. Digital photos from the soil particles with different sizes were used to determine the angularity and sphericity of the particles. Individual particles were selected based on the same method presented in the previous section. The quantitative analysis of 100 soil particles showed that the angularity of the particles fell in the range of 0.18 and 0.48 with the median of 0.34. The sphericity of the particles came into the range of 0.43 and 0.8, with a median of 0.56. The average A and S was 0.34(± 0.07) and 0.58(± 0.1), respectively, which is an indication of sub-angular particle shapes based on the roundness classification scheme presented in Table 1. The average angularity of these particles is towards the upper band of the sub-angular particles (0.25–0.35).

The sub-angular particles from the Micro-Deval test were used to develop soil specimens with the same PSD curve as the angular particles. This was done by sieve analysis, separation of the particles to different sizes and mixing them with various fraction required for the mean PSD curve in Fig. 2.

#### Shear box sample preparation

Direct shear test specimens were prepared at the specific relative densities of 60% and 85%. For instance, maximum and minimum dry density tests were conducted on both samples based on ASTM D4253 and ASTM D4254. The average parameters are presented in Table 3. Gs, γ_d_, γ_dmin_ and γ_dmax_ are specific gravity, dry density, minimum dry density, and maximum dry density of the soil samples in this table.

**Table 3:** Density characteristics of angular and sub-angular material.

Soil type	γ_dmax_ (kN/m^3^)	γ_dmin_ (kN/m^3^)	γ_d (Dr = 60%)_ (kN/m^3^)	γ_d (Dr = 85%)_ (kN/m^3^)	G_s_, –
Angular	16.9	12.4	14.8	16	2.55
Sub-angular	18	14.4	16.4	17.3	2.55

Considering the minimum and maximum relative densities in Table 3 and the specific relative densities, the unit weights of the final material (γ_d_) were determined (Table 3). Based on these dry densities and the initial volume of the shear box, the amount of the material to be used in the shear box was obtained. The soil was compacted within six layers similar to the procedure recommended in ASTM D7181-11^29^, as the “tamping method” to reach the specified density. A square tamper with a cross section of 10 cm × 10 cm was used to compact the material in each layer. This was done via trial and error, so the required materials fitted in the shear box and reached the overall target density. The top layer was compacted with more caution to prepare a horizontal surface for the cap and vertical load. To ensure that the tamping method did not break the particles, the PSD curves of three soil specimens after compaction were determined and compared with those before compaction. The maximum breakage index (BI, introduced in the last section of this article, Particle Breakage) was less than 0.2%, which showed minimal change in the PSD of the soil specimens after compaction.

#### Direct shear test apparatus and testing method

A medium-scale direct shear test apparatus was used in this study. The dimensions of the shear box were 30 cm × 30 cm × 15 cm. The minimum and maximum displacement rate of the equipment was 0.001 and 20 mm/min, respectively, with the shear force capacity of 100 kN. In this study, specimens were subjected to the specified level of normal stresses following a shear load with a constant displacement rate of 1 mm/min.

The direct shear test was conducted in three different states: dry, wet and dry–wet. In the dry condition, the specimen was dry while applying the vertical and shear load. In the wet condition, the specimen was fully submerged during both stages of loading. In the dry–wet condition, the soil specimen was dry during the application of the vertical load and up to a specific shear stress level (SL) during shear loading. Then, at this point, the specimen was submerged from the bottom of the shear box to the top in about 5 min. The specimen was in this state for up to 25 min without any interruption to capture the maximum collapse of the specimen. Finally, the shear load was continued beyond the peak shear stress in the wet state. It should be mentioned that creep deformation was not investigated in this study; however, 25 min after saturation of the samples was sufficient to compare the value of stress release and collapse deformation in different tests.

**Figure 4:**
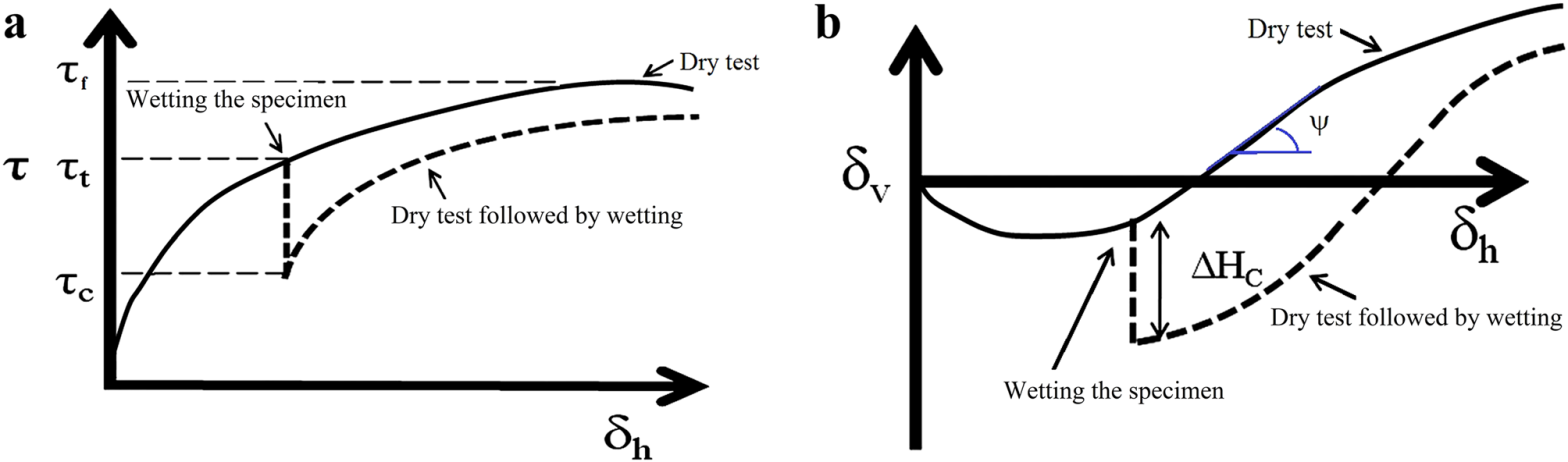
Typical curves for the dry test and dry followed by wetting. (**a**) Shear stress–shear displacement. (**b**) Vertical displacement-shear displacement.

The laboratory study consisted of 40 direct shear tests with the specimens subjected to three vertical pressures of 100, 300 and 500 kPa. The experimental program is presented in Table 4. In this table, δ_h_ and SL are the shear displacement and shear stress level, respectively, immediately before wetting the specimens. SL is the percentage of the current shear stress (τ_t_) to the shear stress at failure point (τ_f_) in the dry condition (Fig. 4a):

1 $$SL=\frac{{\tau_{t} }}{{\tau_{f} }}$$

**Table 4:** Experimental plan.

Purpose of the tests	Shape of the particles	Test type	Relative density (%)	Normal pressure (kPa)	Stress level (SL) during wetting (%)	Test no
Effects of relative density	Angular	Dry–wet	85	100	0	1
				300	0	2
				500	0	3
			60	100	0	4
				300	0	5
				500	0	6
			85	100	50	7
				300	50	8
				500	50	9
			60	100	50	10
				300	50	11
				500	50	12
Effects of normal pressure and stress level	Angular	Dry	85	100	–	13
				300	–	14
				500	–	15
		Wet	85	300	–	16
		Dry–wet	85	100	72 (δ_h_ = 5 mm)	17
				300	46 (δ_h_ = 5 mm)	18
				500	42 (δ_h_ = 5 mm)	19
				300	30	20
				300	50	21
				300	75	22
				300	100	23
				500	50	24
				500	75	25
				500	100	26
	Sub-angular	Dry	85	100	–	27
				300	–	28
				500	–	29
		Wet	85	300	–	30
		Dry–wet	85	100	68 (δ_h_ = 5 mm)	31
				300	54 (δ_h_ = 5 mm)	32
				500	47 (δ_h_ = 5 mm)	33
				300	30	34
				300	50	35
				300	75	36
				300	100	37
				500	50	38
				500	75	39
				500	100	40

SL in soil mass can vary from zero for horizontal layers to 100% in the slopes at the onset of failure. In this study, SLs of 30%, 50%, 75% and 100% are considered for the tests to explore the collapse deformation of the material in a wide range of stress levels, observed in the body of embankment dams^30^ during construction and impoundment or in high road banks.

### Parameters for comparison

Figure 4 shows the typical shear stress–shear displacement and vertical displacement-shear displacement of the direct shear tests in dry and dry–wet conditions. In the latter test, the shear force was applied to the dry samples reaching a shear stress of τ_t_, and then the soil specimen was inundated without shearing for 25 min. After this time, the shear load was continued beyond the peak shear stress. During the inundation of the samples, the collapse behaviour results in a drop in the shear stress to τ_c_ and a sudden vertical displacement called collapse settlement (ΔHc in Fig. 4b). The following parameters are used in this study:

2 $${\tau }_{tc}={\tau }_{t}-{\tau }_{c}$$

3 $${C}_{sr}={\tau }_{c}/{\tau }_{t}$$

where *τ*_*tc*_ and *C*_*sr*_ are stress relaxation and the coefficient of stress recovery, respectively. Equation (3) is similar to the equation suggested by Justo^31^ in the oedometer test. He introduced parameter “a” as the relaxation coefficient with the following relation:

4 $$\text{a}={\tau }_{tc}/{\tau }_{t}=1-{C}_{sr}$$

## Results and discussions

### Collapse settlement, relative density and normal stress level

The purpose of these initial tests was first to confirm the results of the tests with similar studies by other researchers and second, to determine the necessary time for the collapse settlement, which is required in the direct shear tests. The effect of relative density on the collapse settlement of angular material was investigated in two series of tests. In the first series, specimens under specific vertical loads (without shearing) were submerged, and vertical deformation was monitored. In the second series of tests, the inundation of the samples occurred after the vertical load and at a specific shear stress level.

**Figure 5:**
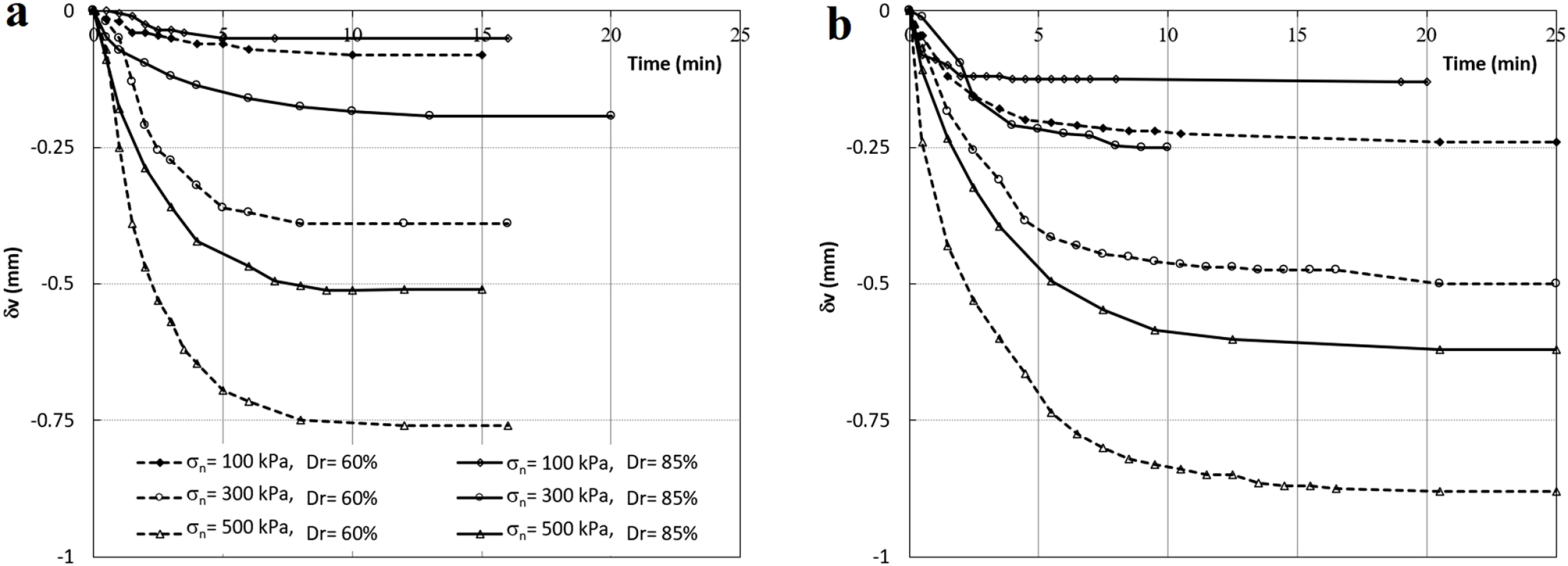
Effect of relative density and normal stress on the collapse settlement. (**a**) SL = 0. (**b**) SL = 50%.

For the first series, six dry samples were prepared in the shear box with relative densities of 60% and 85% and were subjected to vertical loads of 100, 300 and 500 kPa (Table 4, No. 1–6). After reaching the final settlement in the dry state, the samples were submerged, and collapse settlements were measured (shown in Fig. 5a). As can be seen, after 15 min all the samples reach their final settlement. This figure shows that in all vertical stresses, the collapse settlement of the samples decreases with an increase in the relative density. Also, the greater the vertical load, the greater the collapse settlement in the samples. These results confirm the results of the tests conducted by other researchers^16,32,33^.

In the second series of tests, six dry samples were loaded under the same vertical pressures, but the samples were submerged during shear displacement at the shear stress level of 50% (Table 4, No. 7–12). The changes of collapse settlement of the samples with time are shown in Fig. 5b. The variation of collapse settlement with density and vertical pressure is similar to that of previous tests but with higher intensity. It means that the collapse settlement increases with an increase in the shear stress level in all vertical stress and relative densities. These results confirm the finding by Tabibnejad et al.^33^ on rockfills and Hasanzadehshooiili et al.^34^ on coarse and clean gravel.

The above tests showed that the collapse settlement became steady and constant about 25 min after the wetting of the samples, while no shear displacement was applied. Therefore, 25 min was used for the collapse settlement before shearing applied.

### Effect of particle shape on collapse settlement and stress reduction

In total, 28 direct shear tests were conducted with vertical stresses of 100, 300 and 500 kPa and a relative density of 85% at different shear stress levels (Table 4, No. 13–40). This high value of relative density was used because in most large geo-structures (e.g., embankment dams more than 15 m in height), a relative density of 85% is suggested as a desired average density for quality assurance of the compacted shell^35^.

Samples were submerged under two conditions: in specific shear displacement or specific shear stress levels. In test No. 17, 18, 19, 31, 32 and 33 (Table 4), the inundation was made when the samples reached a horizontal displacement of 5 mm. This shear displacement corresponded to a slight change in the shear stress level (from 42 to 72%, Table 4). The rest of the tests were conducted using stress level ranges from 30 to 100% to study the collapse behaviour and shear relaxation of the soil material in a wide range of shear stress levels.

### Collapse settlement and stress reduction due to inundation in a constant shear displacement

**Figure 6:**
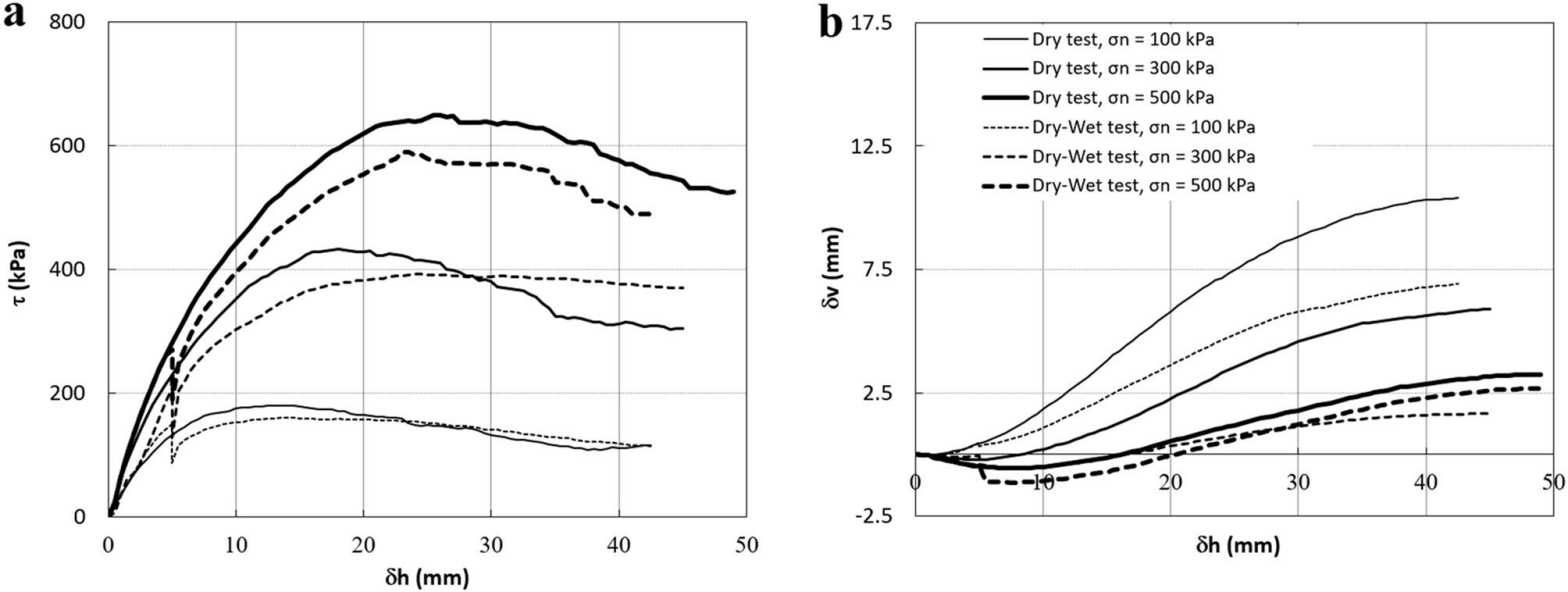
Direct shear test results on specimens with angular particle shapes (Dr = 85%). (**a**) Shear stress–shear displacement. (**b**) Vertical displacement-shear displacement.

**Figure 7:**
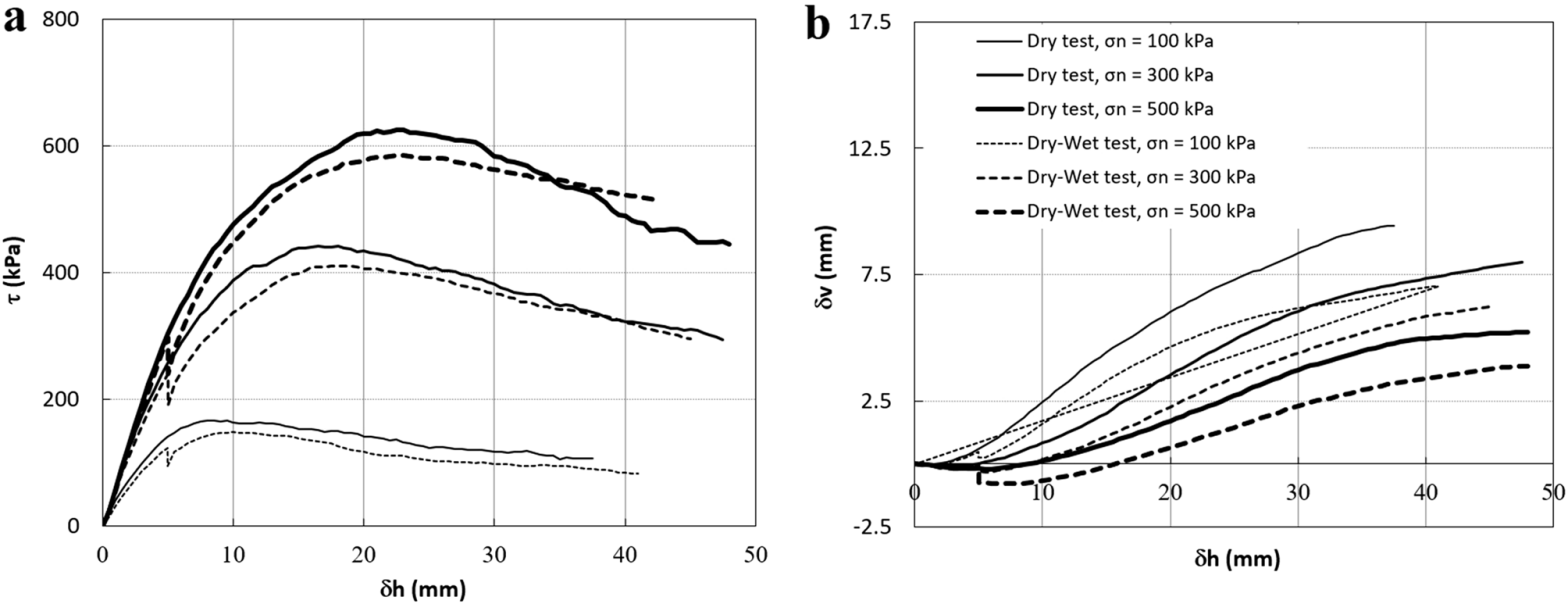
Direct shear test results on specimens with sub-angular particle shapes (Dr = 85%). (**a**) Shear stress–shear displacement. (**b**) Vertical displacement-shear displacement.

For the first series of the tests, Figs. 6 and 7 show the results of the direct shear tests on specimens with angular particle shapes and sub-angular particle shapes, respectively. In these figures, dry tests and dry–wet tests are shown with solid and dashed curves, respectively. Figures 6a and 7a show that the shear strengths of the dry samples are higher than those from the dry–wet tests at the same vertical stresses. This is because samples in the latter tests were inundated before they reached their peak strength. The stress relaxation occurs in all dry–wet tests in both specimens with angular and sub-angular particles. It can be seen that the stress relaxation increases with vertical stress in both types of specimens, which agrees with the trends observed in other coarse grain material such as rockfill material and sandy soils^36,37^.

Figures 6b and 7b show the deformation behaviour of both types of samples subjected to shearing. Compared with the dry–wet samples, dry samples show higher vertical displacement during shearing in both angular and sup-angular particles because of their higher shear strength. Also, it can be seen that an increase in the vertical stress corresponds to a decrease in the dilatancy behaviour in all these tests; the higher the vertical pressure, the higher the degradation in the corners of the particles due to stress concentration^36^. These figures also show that the collapse settlement (ΔH_c_) in both types of samples increases with the vertical stress. This is in agreement with the study by Naderian and Williams^32^ and Heshmati et al.^36^ on other types of granular material.

Another result from the above comparison is that stress relaxation and collapse settlement in specimens with angular particle shapes are higher than those in specimens with sub-angular particle shapes at each level of vertical stress. This is very similar to the study by Shin and Santamarina^21^ on particle angularity and the compressibility of granular mixtures. They showed that with an increase in the fraction of angular particles in the oedometer test, the compression index of the samples increased, resulting in more settlement due to the vertical load.

### Collapse settlement and stress reduction due to inundation in a broader shear stress level

**Figure 8:**
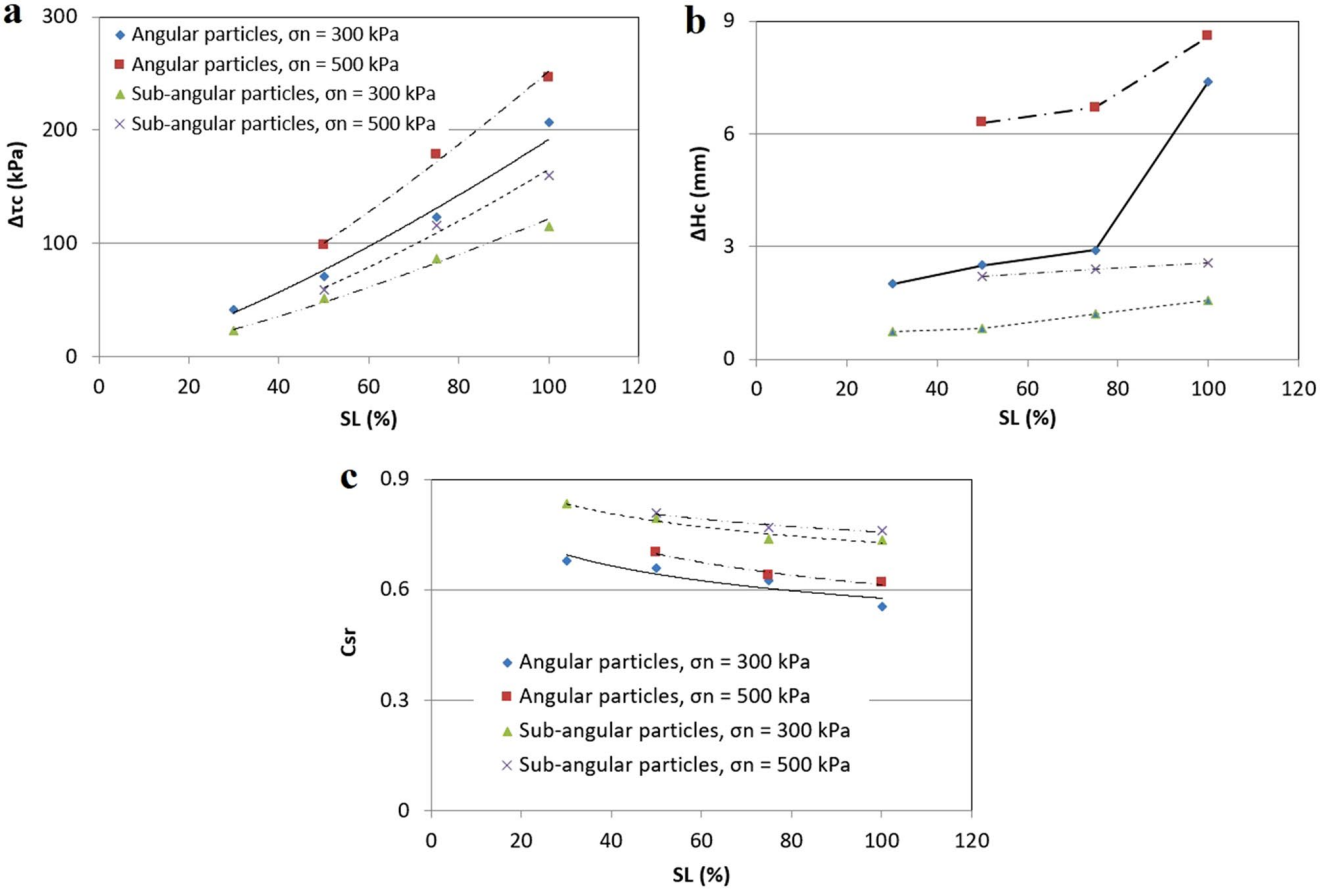
Effect of particle shape on (**a**) stress relaxation, (**b**) collapse settlement and (**c**) coefficient of stress recovery.

Inundation of the samples in the dry–wet tests in the previous section occurred under a specific horizontal displacement of 5 mm, which corresponds to the narrow range in the shear stress levels. To study the collapse behaviour of angular and sub-angular particles in a higher range of shear stress levels, 14 direct shear tests were conducted on initially dry samples with a relative density of 85%, vertical stresses of 300 and 500 kPa and SLs of 30, 50, 75 and 100%. The specifications of these tests are shown in the experimental plan at Table 4 (No. 20–26 and No. 34–40). Based on the results of the shear tests, variation of the stress relaxation, collapse settlement and coefficient of stress recovery to the shear stress levels for vertical stresses of 300 and 500 kPa are shown in Fig. 8.

Figure 8a shows that for both sub-angular and angular materials with an increasing shear stress level and normal pressure, stress relaxation increases. Also, it is evident that material with sub-angular particles in both vertical stresses, shows less stress relaxation than angular particles. This means that dry gravel soils with angular particles are more prone to stress relaxation due to saturation. This reality is associated with more collapse settlement in angular soils, which is illustrated in Fig. 8b. This figure shows changes of collapse settlement with shear stress levels for both angular and sub-angular particles. In both vertical stresses, samples with sub-angular particle shapes show less deformation than those with angular particles. The samples with angular particles are three times more likely to settle than the samples with sub-angular particles. This result is in accord with the study by Alonso^19^ indicating that the collapse settlements of dams made of alluvial gravels with the rounded nature of the particles were very small. The collapse settlement and stress relaxation are higher in higher shear stresses, probably because the stress states in the specimens are closer to the failure line. Higher vertical stress intensifies this phenomenon because there are higher inter-particle stresses.

In Fig. 8c, changes in the coefficient of stress recovery are shown. This figure shows that with an increasing shear stress level, C_sr_ decreases in all types of samples, but this value in sub-angular particles is higher than for angular particles, which shows less collapsibility in the samples with sub-angular particles.

In this study, both types of samples with angular and sub-angular particles have the same relative density and origin, but the material with sub-angular particles is denser than the angular particle material (Table 3). For example, with a relative density of 85%, the weight density of the specimen with sub-angular and angular particles is 1.73 g/cm^3^ and 1.6 g/cm^3^, respectively. This is because the minimum and maximum densities of the soils with sub-angular particles (1.44 and 1.8 g/cm^3^) are higher than those of the soil with angular particles (1.24 and 1.69 g/cm^3^). Both relative density and soil density should be considered in the compaction of the material.

Another reason for the higher value of the collapse settlement in the soil with angular particles is the stress concentration in the corners of the particles in contact with other particles, which results in more rearrangement and particle breakage due to wetting. In soils with angular gravels, particle breakage is paramount for the collapse settlement due to wetting, which is described quantitatively in the following section.

### Particle breakage

**Figure 9:**
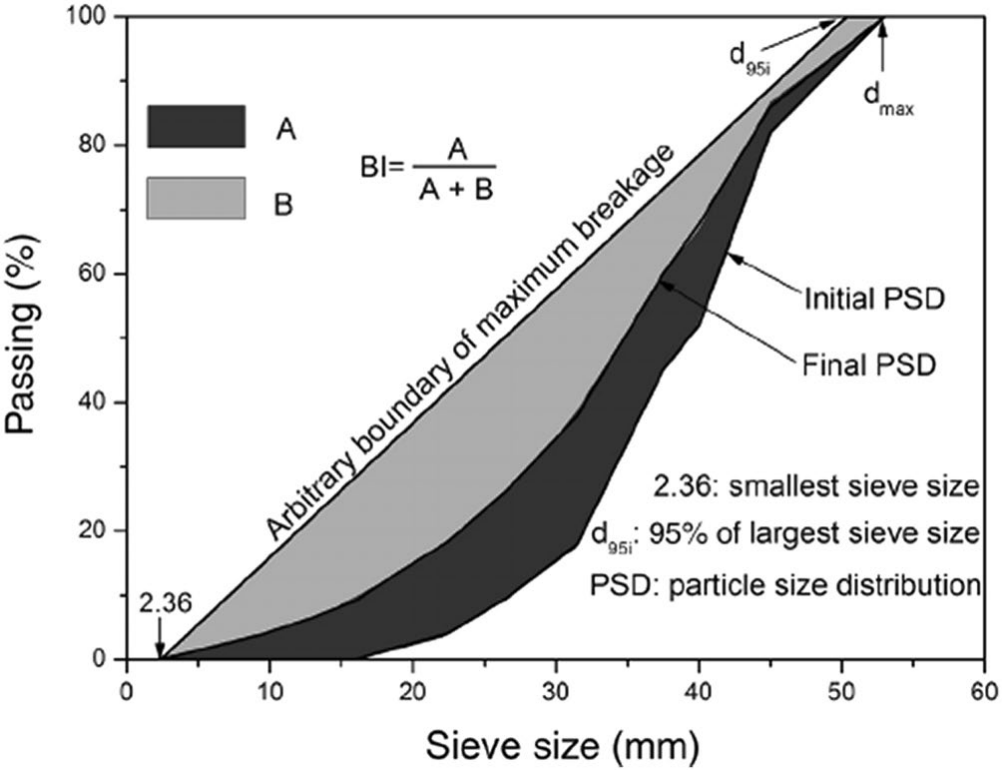
Definition of particle breakage index^38^.

When the stress applied to the soil particle is greater than its strength, the particle may break. As mentioned in the introduction, different factors affect particle breakage. They include the stress level, particle shape and water presence. These factors are the main focus of this section. The sample preparation method, applied loads, and the saturation of the initially dry samples can cause the particles to break during a shear test, resulting in different PSDs at the end of the tests. The intensity of the breakage can be evaluated by comparing the PSDs before and after the test. For instance, the method presented by Indraratna et al.^38^ for the ballast material in railways is used in this section to identify the breakage of the samples quantitatively. In this study, the initial PSD curves of all samples are the same and are shown in Fig. 2. For the final PSD curve, sieve analyses were conducted on the soil material after the shear test. Considering Fig. 9, the breakage index is defined as follows:

5 $$BI = A/\left( {A + B} \right)$$

where A = area between the initial PSD and final PSD and B = area between the final PSD and the arbitrary boundary of maximum breakage. This boundary is a straight line with two specific sieve sizes for zero and 100% passing. In this study, for zero passing, the smallest sieve size of 0.15 mm was used, and for 100% passing, 95% of the largest sieve size (23.75 mm) was used to define the arbitrary boundary. With the above definition, the breakage index is between zero (no breakage) and 100% (maximum breakage).

**Figure 10:**
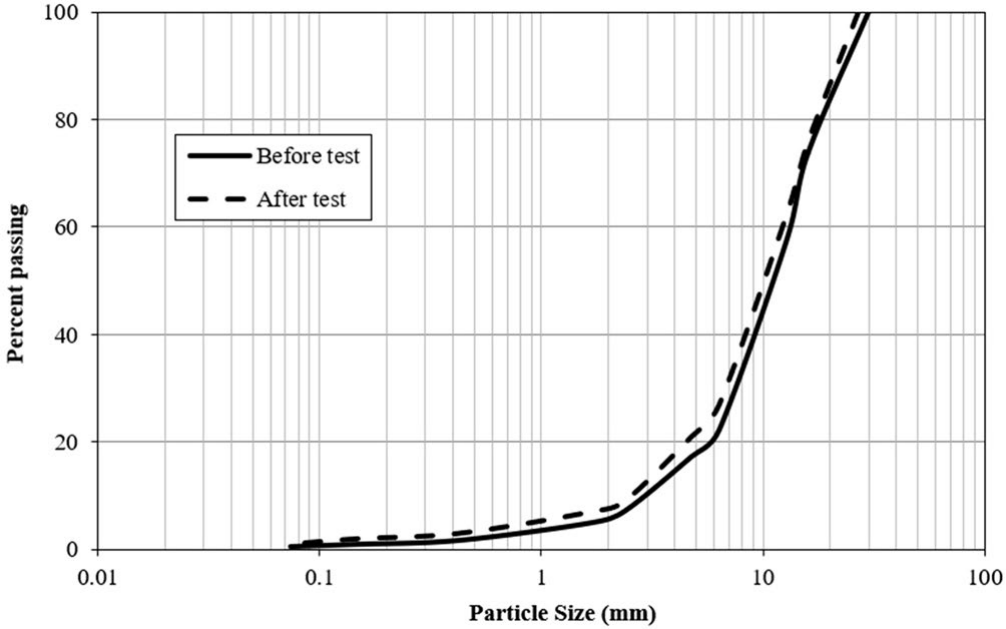
PSD curves before and after the direct shear test on a soil specimen with angular particles (Dry–wet test, σ_n_ = 300 kPa).

By comparing the PSD curves before and after the direct shear tests, the breakage index in 6 soil specimens with a sub-angular particle shape and 4 soil specimens with an angular particle shape was determined in different normal pressures. Figure 10 shows PSD curves for a soil specimen with angular particle shapes. The PSD curve of the soil specimen moved to the left side after the shear test due to particle breakage, resulting in a breakage index of 2.8% based on the above definition.

**Figure 11:**
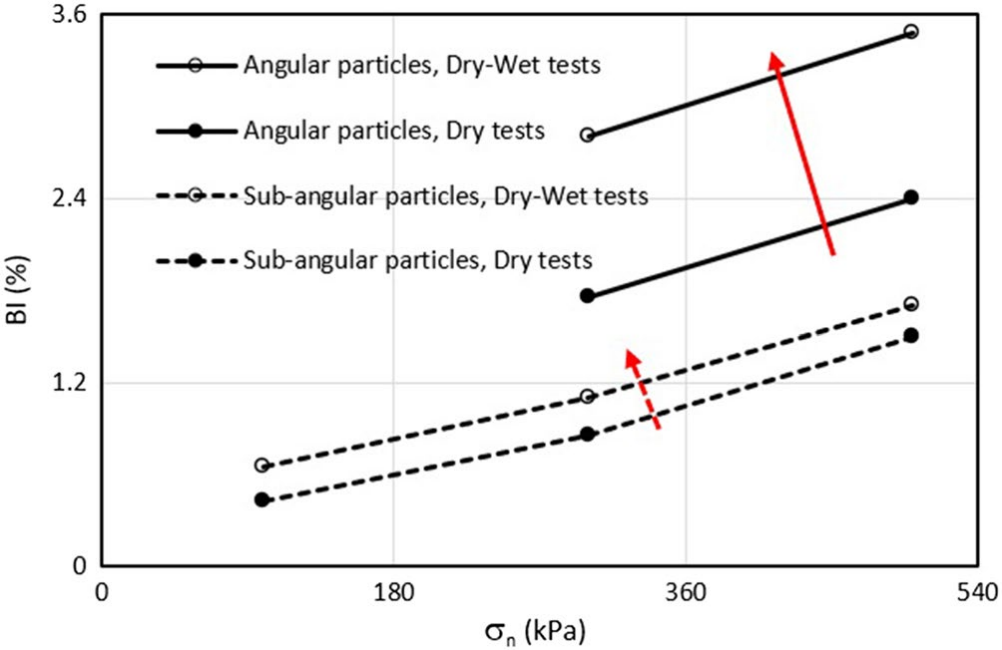
Variation of particle breakage index with normal pressure.

Changes in the breakage index with normal pressure in specimens with angular and sub-angular particle shapes are presented in Fig. 11. As can be seen, the breakage index increases linearly with the normal pressure, which confirms the observations of other researchers^4,36^. Also, the soil specimens with angular particles are more prone to particle breakage during shearing, either in the dry or wet state. This might be associated with higher degradation in the corner of the angular particles due to stress concentration. In both soils with angular and sub-angular particles, the breakage index in dry samples is less than that in the dry–wet test. In dry–wet tests, soil samples were initially dry and then submerged and all the factors (sample preparation, loading and wetting) caused the material to break, while in dry tests, sample preparation and loading were the main factors that caused the breakage of the samples. It can be seen that inundation of the samples has a greater impact on particle breakage in angular particles than sub-angular particles. For example, at the vertical stress of 500 kPa, the BI increases from 2.4 to 3.48 in the sample with angular particles (a 45% increase in BI) in comparison with the increase from 1.5 to 1.7 in the sample with sub-angular particles (a 13% increase in BI).

## Conclusion

This study was designed to investigate the effect of particle shape on the stress reduction and collapse deformation of coarse grain soils due to wetting. A new method was introduced using the current standard procedure for the Micro-Deval test to prepare samples with sub-angular particles from the samples with angular particle shapes. Therefore, the effect of different particle shapes with the same parental rock type, the same PSD curve and the same relative density was studied. A medium-size direct shear test was applied to conduct the shear test on the soil specimens in three different states: dry, wet and dry–wet.

The results showed that the collapse settlement and the stress relaxation increased when the relative density of the soil decreased and the vertical and shear stress increased. The angularity of the particles had a significant influence on the collapse parameters, which needs to be carefully considered when selecting the borrow areas for the embankment dam body or any geotechnical structures. The specimens with angular particle shapes showed a lower coefficient of stress recovery than the specimens with sub-angular particles in all shear stress levels and vertical stresses. It was shown that the shear stress relaxation and collapse settlement in the samples with angular particles were higher than those in the samples with sub-angular particles; the difference increased with vertical stress and shear stress levels.

The main reason for the above phenomenon would be the higher particle breakage in the soil with angular particles due to shearing and wetting. To demonstrate this effect, the particle breakage index was introduced to compare the PSD curves of the soil specimens before and after the direct shear tests. It was shown that the particle breakage index of the samples with angular-particle shapes was higher than for the samples with sub-angular particle shapes. Particle breakage in all samples increased with normal stress and wetting the samples. The effect of wetting on particle breakage of soils with angular particle shapes was significantly higher than for soils with sub-angular particles.
